# A New Metabolism-Related Index Correlates with the Degree of Liver Fibrosis in Hepatitis C Virus-Positive Patients

**DOI:** 10.1155/2015/926169

**Published:** 2015-03-11

**Authors:** Hirayuki Enomoto, Nobuhiro Aizawa, Hideji Nakamura, Ryo Takata, Yoshiyuki Sakai, Yoshinori Iwata, Hironori Tanaka, Naoto Ikeda, Tomoko Aoki, Kunihiro Hasegawa, Kazunori Yoh, Kenji Hashimoto, Akio Ishii, Tomoyuki Takashima, Masaki Saito, Hiroyasu Imanishi, Hiroko Iijima, Shuhei Nishiguchi

**Affiliations:** ^1^Division of Hepatobiliary and Pancreatic Disease, Department of Internal Medicine, Hyogo College of Medicine, Mukogawa-cho 1-1, Nishinomiya, Hyogo 663-8501, Japan; ^2^Department of Gastroenterology and Hepatology, Nissay Hospital, Itachibori 6-3-8, Nishi-ku, Osaka 550-0012, Japan

## Abstract

*Background*. Only a few biomarkers based on metabolic parameters for evaluating liver fibrosis have been reported. The aim of this study was to investigate the relevance of an index obtained from three metabolic variables (glycated albumin: GA, glycated hemoglobin: HbA1c, and branched-chain amino acids to tyrosine ratio: BTR) to the degree of liver fibrosis in hepatitis C virus virus- (HCV-) positive patients. *Methods*. A total of 394 HCV-positive patients were assessed based on the values of a new index (GA/HbA1c/BTR). The index findings were used to investigate the relationship with the degree of liver fibrosis. *Results*. The new index showed an association with the stage of fibrosis (METAVIR scores: F0-1: 0.42 ± 0.10, F2: 0.48 ± 0.15, F3: 0.56 ± 0.22, and F4: 0.71 ± 0.30). The index was negatively correlated with three variables of liver function: the prothrombin time percentage (*P* < 0.0001), albumin level (*P* < 0.0001), and cholinesterase level (*P* < 0.0001). The new index showed a higher correlation related to liver function than FIB-4 and the APRI did. In addition, the index showed a higher AUROC value than that of FIB-4 and the APRI for prediction of liver cirrhosis. *Conclusion*. The new metabolism-related index, GA/HbA1c/BTR value, is shown to relate to the degree of liver fibrosis in HCV-positive patients.

## 1. Introduction

In clinical practice, liver biopsy is the gold standard method to estimate the degree of liver fibrosis [1, 2]. However, the liver biopsy technique is an invasive procedure with a risk of complications. In addition, liver biopsy provides only about 1/50,000 of the organ for the analysis, thus leading to several problems, such as sampling errors, and inter- and intraobserver discrepancies [3, 4]. Noninvasive biomarkers of liver fibrosis have been recently proposed with an evaluation of their clinical utilities [5–7].

The liver is an important metabolic as well as a digestive organ, and metabolic functions can be affected by the progression of liver fibrosis. However, the previously established markers of liver fibrosis available with laboratory tests mainly depend on liver functional tests, such as the values of AST, ALT, gamma-glutamyl transpeptidase (GGT) and platelet count, and only a few biomarkers based on metabolic parameters have been reported.

In patients with chronic liver diseases (CLDs), the level of glycated hemoglobin (HbA1c), which is commonly used as a reliable index of glycemic control in diabetic patients [8, 9], shows lower values relative to the degree of glycemia because of the hypersplenism-associated the abbreviated lifespan of erythrocytes. In contrast, another marker of glycemic control, glycated albumin (GA) [10–13], shows higher values than that expected based on the levels of glycemia, because the turnover of serum albumin is prolonged in order to compensate for the decreased albumin production in the liver. Based on the fact that the HbA1c shows lower and the GA shows higher values in CLD patients, we have reported that the GA/HbA1c ratio was increased in line with the histological severity of the liver fibrosis in various CLDs [14–17], even before the fibrosis progressed to cirrhosis. Although the GA/HbA1c ratio was a unique biomarker independent of the common liver functional tests, such as AST, ALT, and platelet count, we found that the ratio differences among the fibrotic stages were relatively small. This suggested that a more sensitive index was needed.

Recently, we have focused on another metabolic variable, the amino acid imbalance, in CLD patients. We investigated the branched chain amino acids- (BCAA-) to-tyrosine ratio (BTR) value which is an inexpensive and easily measurable index of the amino acid imbalance [18, 19]. We found that the BTR value was decreased as liver fibrosis progressed in patients infected with hepatitis C virus (HCV) [20]. In the present study, we combined these metabolic-related markers (the GA/HbA1c ratio and the BTR value) and investigated the association of these combined markers with the histological stage of liver fibrosis in HCV-positive patients. Furthermore, we assessed the diagnostic performance of the new biomarker and compared it to the other well-known fibrosis biomarkers.

## 2. Patients and Methods

### 2.1. Patients

We studied a total of 394 HCV-positive patients who had undergone percutaneous liver biopsies betweenJanuary 2009 and July 2013 at our institution. This study consecutively included all patients who fulfilled the following conditions: HCV infection was diagnosed by the detection of HCV antibodies and HCV-RNA in serum. Blood samples, including samples for an analysis of the GA, HbA1c, and BTR levels, were obtained on the same day as the liver biopsies. Patients with the following conditions were excluded from the study: the presence of other liver diseases, hepatocellular carcinoma, immunosuppressive therapy, coinfection of hepatitis B virus (HBV), and those with insufficient liver tissue for the staging of fibrosis (a minimum of 15 mm of liver tissue with five or more portal tracts is required for diagnosis). The present study did not include patients whose GA/HbA1c ratios could have been influenced by poorly controlled diabetes. We also excluded patients who received BCAA treatment because of its increasing effect on the serum BTR value. The characteristics of the study population are summarized in Table 1. The study conformed to the ethical guidelines of the declaration of Helsinki, and written informed consent regarding the liver biopsy and use of clinical data was obtained from all patients on admission. This study was approved by the ethics committee of the institutional review board.

### 2.2. Liver Biopsy and Laboratory Data

Liver biopsy samples were obtained using standard methods, and well-trained pathologists at our institute evaluated the samples based on fibrotic stage and activity grade. Fibrosis was evaluated according to the METAVIR scoring system [21] and was staged on a scale of F0–F4 (F0, no fibrosis; F1, portal fibrosis without septa; F2, portal fibrosis with rare septa; F3, numerous septa without cirrhosis; F4, liver cirrhosis). The histological findings of the biopsy tissues were also routinely evaluated in our department. All authors participated in the conferences on the histological findings and the final results were confirmed by all authors.

HbA1c was measured by high-performance liquid chromatography, with a calibration as recommended by the Japan Diabetes Society (JDS) Lot 2 [22]. The value for HbA1c (%) was estimated as a NGSP equivalent value (%) calculated using the following formula: in the range of JDS values ≤4.9%: NGSP (%) = JDS (%) + 0.3% and in the range of JDS 5.0–9.9%: NGSP (%) = JDS (%) + 0.4% [23]. BTR values were measured using a commercially available kit (Daiyacolor-BTR, Toyobo, Osaka, Japan) [18]. Routine laboratory studies, including platelet counts, the prothrombin time (PT) percentage, and other liver function tests, such as ALT, AST, alkaline phosphatase, albumin, and cholinesterase, were also performed.

We previously reported that the mean value of the GA/HbA1c ratio increased as the degree of liver fibrosis increased. In contrast, the mean value of BTR was decreased as the degree of liver fibrosis increased. Based on the increased GA/HbA1c value and the decreased BTR value, we calculated the ratio of these two parameters in order to amplify the changes observed during the progression of liver fibrosis. Therefore, the value of GA/HbA1c/BTR (=[GA/HbA1c ratio]/BTR) was determined as a new metabolism-related index. Here we examined the association of the new index with the histological stage of liver fibrosis.

In the present study, the values of two highly used biomarkers for liver fibrosis (FIB-4 and the APRI: the AST-to-platelet count ratio index) were also calculated based on the following formulas: FIB-4 = Age [years] × AST [U/L]/(platelets [10^9^/L] × (ALT [U/L])^1/2^), in which the age of the patient is the age at the time of liver biopsy [24, 25], and APRI = 100 × (AST level/upper limit of normal)/platelets [10^9^/L] [26].

### 2.3. Statistical Analysis

In the present study, we investigated whether the new index (GA/HbA1c/BTR) is associated with the degree of liver fibrosis in HCV-positive patients. The data for the comparisons among the groups “F0-1 versus F2 versus F3 versus F4” were analyzed by non-repeated measurements ANOVA, and statistical significance was consequently evaluated with the Bonferroni correction. The association of the new index with other variables, including FIB-4 and APRI, was evaluated with Spearman's correlation coefficient. A value of *P* < 0.05 was considered to be significant.

The diagnostic values of three markers (GA/HbA1c/BTR, APRI, and FIB-4) were assessed by calculating the area under the receiver operating characteristic curves (AUROC) with the determination of the cut-off values. Diagnostic performance was evaluated by sensitivity, specificity, positive predictive value (PPV), and negative predictive value (NPV). Statistical analysis was performed with the JMP 9 (SAS Institute Inc., Cary, NC, USA).

## 3. Results

### 3.1. Metabolic-Related Markers Are Associated with the Histological Stage of HCV-Positive Patients

A total of 394 HCV-positive patients were evaluated, and the characteristics of the enrolled patients are summarized in Table 1. The population consisted of 181 (45.9%) male patients and 213 (54.1%) female patients, and the age of patients ranged from 25 to 85 years (median 61 years). The Child-Pugh score classified all cirrhotic patients (F4 stage) as grade A. In agreement with our previous reports, the histological severity of liver fibrosis in the HCV-positive patients significantly correlated with the increased GA/HbA1c ratio and the decreased BTR value (Table 2).

**Figure 1:**
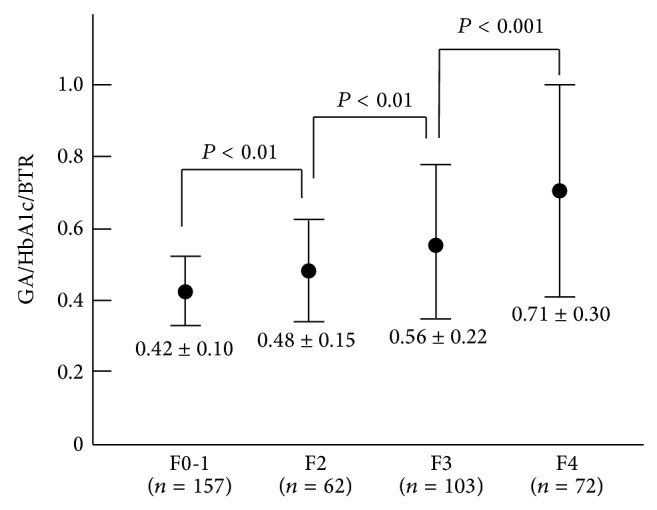
Values of the metabolic-related new index (GA/HbA1c/BTR) in HCV-positive patients. The GA/HbA1c/BTR value increase correlated with the stage of liver fibrosis. There were significant differences in all of the comparisons between the fibrotic stages, including F0-F1 versus F2, F2 versus F3, and F3 versus F4 groups.

As described in Section 2, we combined these variables in order to amplify the changes observed during the progression of liver fibrosis and thus defined a new metabolism-related index (GA/HbA1c/BTR). The newly determined metabolism-related index significantly correlated with the histological stage of liver fibrosis (*P* < 0.0001), and the levels of the new index were significantly different in all comparisons between the fibrotic stages, including F0-1 versus F2, F2 versus F3, and F3 versus F4 (Figure 1). Since the new index was related to the histological stage of liver fibrosis in the HCV-positive patients, we investigated whether the index correlated with two well-established biomarkers for liver fibrosis, FIB-4 and APRI. The new index significantly correlated with both FIB-4 (*R* = 0.643, *P* < 0.0001) and the APRI (*R* = 0.445, *P* < 0.0001). These findings suggest a significant association of the new index with the histological severity of liver fibrosis.

### 3.2. The Metabolism-Related Index Is Associated with Both Decreased Liver Function and Cirrhotic Change in HCV-Positive Patients

We next investigated whether the new index correlated with the laboratory parameters of the liver function, including the albumin and cholinesterase levels and PT percentage. The index reciprocally correlates with the serum albumin level (*R* = −0.575, *P* < 0.0001), the cholinesterase level (*R* = −0.589, *P* < 0.0001), and the PT percentage (*R* = −0.481, *P* < 0.0001) in HCV-positive patients (Table 3). The new index showed a higher correlation with these three parameters of liver function tests (albumin value, cholinesterase value, and PT percentage) than that of either the GA/HbA1c alone or the BTR alone. Furthermore, the new index also showed a higher correlation with these three parameters than that of the FIB-4 and the APRI (Table 3). When we plotted the ROC curves of the three markers (GA/HbA1c/BTR, FIB-4, and APRI) for the prediction of severe fibrosis (≥F3), the AUROC value of the new index (0.744) was higher than that of the APRI (0.722), but lower than that of FIB-4 (0.753). However, the AUROC value of the new index was the highest among the three biomarkers for the prediction of liver cirrhosis (F4) (Table 4). In addition, the new index showed the highest NPV among the markers, suggesting its positive diagnostic performance to discriminate the HCV-positive noncirrhotic patients from cirrhotic patients.

## 4. Discussion

Liver biopsy has been generally accepted as the most reliable method for evaluating the degree of liver fibrosis. However, a liver biopsy is an invasive burden for patients, and it is not easy for the patient to have this procedure frequently to follow the disease progression. Recently, it has been shown that imaging waves can provide a new noninvasive assessment for liver fibrosis [5–7], and in particular, transient elastography (TE) [27, 28] is widely used as a diagnostic tool. Although TE is an excellent noninvasive and reproducible method, it is difficult to apply to patients with narrow intercostal spaces and/or with severe obesity. In addition, special imaging devices are expensive and difficult to routinely use in the daily clinical practice of every institute. Therefore, noninvasive biomarkers are attractive in that they can be easily applied to all patients, since only blood samples are required. In addition to the FIB-4 and APRI, noninvasive biomarkers, such as the Fibro-Test score [29], Forns score [30], Fibrosis Probability Index [31], Hepascore [32], FibroMeter [33], Lok index [34], Fibro Index [35], Enhanced Liver Fibrosis score [36], and Fibrospect [37], were reported to be significantly associated with the degree of liver fibrosis. Most of these previously established biomarkers are obtained based on calculations that include serum biochemical variables such as AST, ALT, and GGT values. However, even in the same patient, these values can vary depending on when the blood sample is collected. Furthermore, recent progress in antiviral treatments has normalized the values of AST and ALT in many patients [38], even though the patients still have fibrotic liver. We have therefore attempted to find a biomarker which is independent of these parameters. We have focused on metabolism-relate variables to assess the degree of liver fibrosis, because the liver functions as a major metabolic organ as well as a digestive system organ and the metabolic dysfunctions due to the progression of liver disease should be present in CLD patients after normalizing the values of transaminases.

With regard to the metabolism-related biomarkers to assess liver fibrosis, some groups, including ours, reported that the GA/HbA1c ratio was associated with the degree of liver fibrosis in various types of CLD, such as HCV-related CLD, HBV-related CLD, and nonalcoholic steatohepatitis [14, 16, 17, 39]. Additionally, we have reported that the BTR value was associated with the degree of liver fibrosis and that the severity of esophageal varies in HCV-positive patients [20]. Furthermore, a recent report by Eslam et al. [40] also showed the possibility of predicting portal hypertension using three metabolic parameters, thus suggesting that metabolism-related parameters could be potential biomarkers for the severity of CLD.

Although we previously showed that the GA/HbA1c ratio was significantly related to the histological stage of liver fibrosis, its diagnostic performances was relatively low when it was used as a sole biomarker [14]. Based on our recent finding showing a significant correlation of the BTR value with the degree of liver fibrosis, we combined these markers (the GA/HbA1c ratio and the BTR value) and defined a new index. We showed that the value of the new index increased with the progression of fibrosis, thus suggesting that the combination can be a unique marker of liver fibrosis and stage (Figure 1).

Among the previously reported biomarkers for liver fibrosis, the FIB-4 and APRI indices are excellent markers that are calculated easily based on a few routine clinical variables. Using the three parameters of the liver function test, we found that the correlation coefficients of the new index were higher than those of both the FIB-4 and the APRI (Table 3). Additionally, for the prediction of cirrhosis, the new index showed a higher AUROC value than that of both FIB-4 and the APRI (Table 4). Although our results do not indicate the superiority of the new index to other biomarkers, our results at least suggest that the new index can be a potential biomarker of liver fibrosis and stage of fibrosis in HCV-positive patients. Although we did not evaluate the index in healthy controls, it would be interesting to investigate whether HCV-positive patients with minimal fibrosis (F0-1) have lower values than healthy controls.

In the present study, we simply divided the GA/HbA1c ratio by BTR and defined the calculated value as a new index. The new index showed higher correlation with the three parameters of liver function tests (PT percentage, albumin value, and cholinesterase value) than those of either the GA/HbA1c alone or the BTR alone (Table 3). However, the combination may not be the final optimal index, and we may search for a better index with another algorithm of these parameters. In developing a better index, one needs to remember that a complex calculation would be inconvenient for wide spread clinical use. In addition, another combination with several parameters may provide a better marker. For instance, the platelet count of a patient does not change in a short period even after the normalization of AST and ALT by antiviral treatment, and the combination of the platelet count with metabolic parameters may be a new biomarker.

In summary, we showed that a new index, which depends on the metabolic changes in CLD patients, showed increases correlating with the stage of liver fibrosis and correlated with the levels of liver fibrosis-related markers in HCV-positive patients.

## Figures and Tables

**Table 1:** Characteristics of the 394 hepatitis C virus- (HCV-) positive patients.

Age (years)	61 (25–85)
Gender (male/female)	181/213
AST (IU/L)	38 (10–385)
ALT (IU/L)	37 (7–725)
*γ*-GTP (IU/L)	29 (5–446)
ALP (IU/L)	217 (97–783)
Total bilirubin (mg/dL)	0.8 (0.2–2.5)
Albumin (g/dL)	3.90 ± 0.37
Hemoglobin (g/dL)	13.6 ± 2.3
Platelets (×10^3^/*μ*L)	162 ± 72
Prothrombin time (%)	89.2 ± 12.2
Diabetes mellitus (present/absent)	36/358
Glucose (mg/dL)	98.2 ± 10.6
Triglyceride (mg/dL)	99.9 ± 46.2
Total cholesterol (mg/dL)	168 ± 36
Body mass index	22.9 ± 3.6
Histological stage of liver fibrosis (F0-1/F2/F3/F4)	157/62/103/72

**Table 2:** Changes in both the GA/HbA1c and the BTR values in HCV-positive patients with histological stage of liver fibrosis.

	Histological stage of liver fibrosis F3				*P* value
	F0-1	F2	F3	F4	
GA/HbA1c	2.61 ± 0.28	2.62 ± 0.30	2.77 ± 0.38	2.96 ± 0.47	<0.0001
BTR	6.38 ± 1.18	5.78 ± 1.33	5.40 ± 1.35	4.63 ± 1.25	<0.0001

GA/HbA1c: glycated albumin- (GA-) to-glycated hemoglobin (HbA1c) ratio.

BTR: branched-chain amino acids to tyrosine ratio.

**Table 3:** Correlation of the three biomarkers with liver function parameters in HCV-positive patients.

	Correlation coefficient				
	GA/HbA1c/BTR	GA/HbA1c	BTR	FIB-4	APRI
Prothrombin time (%)	−0.481	−0.456	0.368	−0.439	−0.328
Albumin value	−0.574	−0.443	0.462	−0.528	−0.423
Cholinesterase value	−0.589	−0.561	0.502	−0.546	−0.456

GA/HbA1c: glycated albumin- (GA-) to-glycated hemoglobin (HbA1c) ratio.

BTR: branched-chain amino acids to tyrosine ratio.

APRI: AST-to-platelet ratio index.

**Table 4:** Comparison of three biomarkers for the prediction of liver cirrhosis.

	ACUROC	Sensitivity	Specificity	PPV	NPV
GA/HbA1c/BTR (≥0.548)	0.782	70.8%	76.7%	40.5%	92.2%
APRI (≥1.14)	0.732	65.3%	72.9%	35.1%	90.4%
FIB-4 (≥1.17)	0.779	66.7%	82.0%	45.3%	91.7%

AUROC: area under the receiver operating characteristic curves.

PPV: positive predictive value.

NPV: negative predictive value.

## Abbreviations

GGT: Gamma-glutamyl transpeptidase
CLD: Chronic liver disease
HbA1c: Glycated hemoglobin
GA: Glycated albumin
BCAA: Branched chain amino acids
BTR: Branched chain amino acids-to-tyrosine ratio
HCV: Hepatitis C virus
HBV: Hepatitis B virus
PT: Prothrombin time.
